# Nitrogen flow boiling and chilldown experiments in microgravity using pulse flow and low-thermally conductive coatings

**DOI:** 10.1038/s41526-022-00220-9

**Published:** 2022-08-09

**Authors:** Jason Hartwig, J. N. Chung, Jun Dong, Bo Han, Hao Wang, Samuel Darr, Matthew Taliaferro, Shreykumar Jain, Michael Doherty

**Affiliations:** 1 NASA Glenn Research Center, Cleveland, OH 44135 USA; 2 University of Florida, Gainesville, FL 32611 USA; 3 The Aerospace Corporation, El Segundo, CA 90245 USA; 4 Georgia Tech University, Atlanta, GA 30332 USA

**Keywords:** Aerospace engineering, Mechanical engineering, Mechanical properties

## Abstract

The enabling of in-space cryogenic engines and cryogenic fuel depots for future manned and robotic space exploration missions begins with technology development of advanced cryogenic fluid management systems upstream in the propellant feed system. Before single-phase liquid can flow to the engine or customer spacecraft receiver tank, the connecting transfer line must first be chilled down to cryogenic temperatures. The most direct and simplest method to quench the line is to use the cold propellant itself. When a cryogenic fluid is introduced into a warm transfer system, two-phase flow quenching ensues. While boiling is well known to be a highly efficient mode of heat transfer, previous work has shown this efficiency is lowered in reduced gravity. Due to the projected cost of launching and storing cryogens in space, it is desired to perform this chilldown process using the least amount of propellant possible, especially given the desire for reusable systems and thus multiple transfers. This paper presents an assessment of two revolutionary new performance enhancements that reduce the amount of propellant consumed during chilldown while in a microgravity environment. Twenty-eight cryogenic transfer line chilldown experiments were performed onboard four parabolic flights to examine the independent as well as combined effect of using low thermally conductive coatings and pulse flow on the chilldown process. Across a range of Reynolds numbers, results show the combination significantly enhances performance in microgravity, with a reduction in consumed mass up to 75% relative to continuous flow for a bare transfer line.

## Introduction

The enabling of in-space cryogenic engines and cryogenic fuel depots for future manned and robotic space exploration missions begins with technology development of advanced cryogenic fluid management (CFM) systems upstream in the propellant feed system. Cryogenic propellants offer significantly higher performance relative to storable counterparts, such as hydrazine, owing to a higher specific impulse and higher energy density. Further, safety and environmental concerns over the use of toxic storable propellants have led to the ongoing examination of more “green” propellants such as liquid methane as alternate fuel sources. Aside from nuclear thermal propulsion systems^1^, no other known pure chemical propulsion system propellant combination can deliver a higher ISP than liquid hydrogen/liquid oxygen. However, there are challenging aspects when working with cryogens due to inherent thermo-physical properties. Particularly for the current work, the low normal boiling point (NBP), low surface tension, and high susceptibility to parasitic heat leak leads to unwanted boiling and two-phase flow during propellant transfer.

Cryogenic fuel depots^2,3^, defined as an Earth-orbiting propellant storage vessel that would house cryogenic propellant to allow spacecraft to refuel, have four stages: (1) acquisition of the storage tank liquid, (2) chilldown of the connecting transfer line hardware, (3) chilldown of the receiver tank, and (4) fill of the receiver tank, all in the microgravity of space; this paper focuses on the second stage, chilldown of the transfer line. Meanwhile, cryogenic engines also require acquisition of the storage tank liquid and chilldown of the transfer line. Cryogenic fuel depots will require very high liquid volume fill fractions in the customer receiver tank, and most in-space engines require single-phase liquid up to the injectors. Therefore, without advanced CFM technologies upstream in the feed system and storage tank, vapor ingestion is inevitable, which can lead to combustion instabilities within the engine. Further exacerbating the transfer process in microgravity is the unknown location of the liquid and vapor phases in the tank as well as reduced heat transfer.

Before single-phase liquid can flow to the engine or customer spacecraft receiver tank, the connecting transfer line must first be chilled down to cryogenic temperatures. Chilldown, or quenching, is defined as the transient process of cooling hardware down to cryogenic temperatures so that vapor-free liquid can eventually flow between two points of interest. The most direct and simplest method to quench the line is to use the cold propellant itself. When a cryogenic fluid is initially transferred through a system, the tube walls and hardware (e.g. valves) undergo a transient chilldown prior to reaching a steady state of operation. Chilldown thus involves unsteady two-phase heat and mass transfer and flow boiling. While boiling is well known to be a highly efficient mode of heat transfer, previous work has shown this efficiency is significantly lowered in reduced gravity, both for room temperature fluids as well as cryogens^4–8^. Due to the projected cost of launching and storing cryogens in space, it is desired to perform this chilldown process using the least amount of propellant as possible, especially given the drive towards reusable systems and thus multiple transfers.

Numerous cryogenic flow boiling quenching experiments have previously been conducted on bare tubes, the results up to 2018 of which are summarized in ref. ^9^. Key contributions in 1-g were provided by^4,5,10–23^, which investigated the effect of mass flux, inlet state, pressure, and flow direction on cryogenic tube chilldown, predominately using liquid nitrogen (LN_2_) and liquid hydrogen (LH_2_). Since 2018, five more cryogenic quenching studies on bare tubes that passed the data filtering criteria from ref. ^9^ by Jin et al. have been added to the cryogenic database to cover low Reynolds (Re) number LN_2_^24^, liquid argon^25^, and liquid oxygen^26^ chilldown experiments while^27,28^ added high Re number chilldown tests with LH_2_. Hartwig et al.^29^ recently summarized cryogenic quenching flow boiling trends over the consolidated literature, across multiple flow regimes, mass fluxes, inlet states, and gravity levels. For all cryogens, the chilldown process is highly dominated by the film boiling regime for bare tubes^30^ (for quantum fluids such as hydrogen and helium, there are additional factors at play). When a cryogen is introduced into a warm tube, especially at high mass flux and low inlet equilibrium quality, a vapor film blanket surrounds the liquid core which acts as an insulator that inhibits heat transfer between cold liquid and warm tube. At lower mass flux and saturated inlet states, dryout occurs over a longer distance along the tube as in the case of traditional fluids^31^. Film boiling heat transfer is a highly inefficient process relative to transition and nucleate boiling. In most instances, film boiling can persist for >85% of the total time needed to chill the tube down to the saturation temperature of the cryogen. Once the Leidenfrost point is reached, chilldown proceeds into transition boiling, nucleate boiling, and then single-phase liquid convective flow. In microgravity, this poor heat transfer is exacerbated by the lack of buoyancy force; cryogenic film boiling heat transfer was shown to be 25% lower at low to modest Re flows relative to 1-g^8^. At very high Re, inertial forces can overcome gravitational forces such that gravity no longer affects flow boiling^32^ (although this has not been demonstrated yet for cryogens).

To overcome this hurdle in poor performance, researchers have recently investigated low thermally conductive materials applied to the inner tube walls and the effect of such coatings on the chilldown process. The coating acts as an insulator between the cold propellant and warm wall, resulting in an inner wall surface temperature that reaches the Leidenfrost point without cooling the entire tube mass. Recent 1-g experiments conducted in the United States^33,34^ and China^35^ independently confirmed that a Teflon coated tube could reduce chilldown times up to 75% over an uncoated stainless steel (SS) tube using LN_2_. Both researchers also investigated the effect of the Teflon coating thickness on chilldown performance, and both showed that thicker coatings led to faster chilldown times. However, as the coating thickness increased further there was an apparent point of diminishing returns because the chilldown curves (wall temperature versus time) converged at the highest tested thicknesses. Coated tubes offer hope to combat the intrinsically poor film boiling heat transfer in microgravity^36^.

A second way to enhance poor chilldown performance is to use pulse flow. Demonstrated using both LH_2_^37^ and LN_2_^38^, in pulse flow, the inlet valve is cyclically opened and closed with a specified duty cycle (DC) and pulse width until the desired degree of chilldown is reached. The advantage of pulse flow is lower mass consumption over traditional continuous flow due to more efficient usage of latent and sensible energy of the fluid, with the disadvantage being potential valve fatigue and/or failure and added complexity in operation.

The purpose of this paper is to present an assessment of two new performance enhancements that reduce the amount of propellant consumed during chilldown while in a microgravity environment, and to investigate if the mass savings holds in microgravity. Twenty-eight LN_2_ transfer line chilldown experiments were performed onboard a parabolic flight that simulated space microgravity conditions to examine the independent as well as combined performance gains of using low thermally conductive coatings and pulse flow on the chilldown process. While previous experiments have reported the effects of pulse flow and coatings on transfer line chilldown in Earth-gravity, this is the first report of pulse flow and the combined effect of pulse flow with a coated tube in a microgravity environment.

## Results and discussion

### Test matrix

Table 1 lists the complete flight test matrix. Ground tests were performed at the University of Florida, while flight tests were conducted during the low-gravity portion of the classic parabolic trajectory followed by the flight provider ZeroG. Pressure is the measured pressure at the inlet to the test section, time-averaged over the test duration. Period is the sum of valve “on” and “off” time for a pulse flow test cycle. The duty cycle is the ratio of the valve “on” time to the period. For example, with a period of 3 s and a duty cycle of 10%, the valve is on 0.3 s and off for 2.7 s. G level is the gravity level as read by accelerometers attached to the experimental rig while on the flight. Note that a few of the flight tests were conducted at a g-level higher than nominal; these were deemed “Martian gravity tests”. Coating thickness in number of layers, “L”, is described in the “Methods” section.

**Table 1:** Flight test matrix.

#	Test name	Inlet pressure	Period [s]	Duty cycle [%]	G Level [g/g_0_]	Coating thickness	Chilldown time [s]	Total chilldown mass [kg]	Steady-state Re	Steady-state mass flux [kg/m^2^-s]
F1	Flight_day1_1	550 kPa [80 psia]	–	100	0.38	Bare	15.1	1.08	71,976	548.87
F2	Flight_day1_2	550 kPa [80 psia]	–	100	0.05	Bare	14.6	0.872	67,324	533.22
F3	Flight_day1_3	550 kPa [80 psia]	2	10	0.05	Bare	16	0.597	53,859	426.58
F4	Flight_day1_4	550 kPa [80 psia]	3	10	0.05	Bare	16.7	0.614	64,092	507.63
F5^a^	Flight_day1_5	340 kPa [50 psia]	–	100	0.05	Bare	70.6	0.264	1761	22.97
F6^a^	Flight_day1_6	340 kPa [50 psia]	4	10	0.05	Bare	78.1	0.279	1383	18.04
F7	Flight_day2_1	550 kPa [80 psia]	3	7	0.38	Bare	21.3	0.637	32,621	347.47
F8^a^	Flight_day2_2	340 kPa [50 psia]	–	100	0.05	Bare	80.3	0.279	1566	20.42
F9^a^	Flight_day2_3	340 kPa [50 psia]	5	4	0.05	Bare	162	0.304	881	11.49
F10^a^	Flight_day2_4	340 kPa [50 psia]	–	100	0.05	Bare	172	0.16	992	15.94
F11^b^	Flight_day2_5	440 kPa [65 psia]	3	7	0.05	Bare	21.3	0.645	29,761	306.99
F12^b^	Flight_day2_6	440 kPa [65 psia]	–	100	0.05	Bare	21.1	1.174	53,021	459.65
F13	Flight_day3_1	550 kPa [80 psia]	–	100	0.05	7 L	8.19	0.469	63,912	506.19
F14	Flight_day3_2	550 kPa [80 psia]	3	7	0.05	7 L	9.94	0.339	38,308	351.49
F15	Flight_day3_3	550 kPa [80 psia]	–	100	0.05	7 L	6.69	0.309	45,760	340.84
F16	Flight_day3_4	550 kPa [80 psia]	3	7	0.05	7 L	10	0.248	43,178	329.21
F17	Flight_day3_5	340 kPa [50 psia]	–	100	0.05	7 L	12.8	0.0881	8945	73.36
F18	Flight_day3_6	440 kPa [65 psia]	3	7	0.05	7 L	9.31	0.283	38,265	364.8
F19^c^	Flight_day3_7	440 kPa [65 psia]	–	100	0.05	7 L	7.06	0.514	83,353	649.18
F20	Flight_day3_8	440 kPa [65 psia]	–	100	0.05	7 L	7.88	0.585	88,127	682.68
F21	Flight_day3_9	340 kPa [50 psia]	–	100	0.05	7 L	12.8	0.0855	10,949	90.82
F22^d^	Flight_day4_1	550 kPa [80 psia]	–	100	0.05	4 L	4.69	0.281	74,196	558.16
F23^d^	Flight_day4_2	550 kPa [80 psia]	3	7	0.05	4 L	9.06	0.269	46,373	348.85
F24^d^	Flight_day4_3	550 kPa [80 psia]	2	10	0.05	4 L	6.19	0.237	63,067	474.44
F25^d^	Flight_day4_4	440 kPa [65 psia]	–	100	0.05	4 L	4.5	0.23	59,931	476.14
F26^d^	Flight_day4_5	440 kPa [65 psia]	3	7	0.05	4 L	10	0.288	50,941	404.72
F27^d^	Flight_day4_6	340 kPa [50 psia]	–	100	0.05	4 L	7.81	0.0555	8061	66.22
F28^d^	Flight_day4_7	550 kPa [80 psia]	3	10	0.05	4 L	7.25	0.274	64,048	500.43

^a^These cases were conducted over multi-parabolas; the calculations also include the high G part.

^b^Airplane accelerated to 1 g before end of chilldown.

^c^The initial temperature is lower than other cases because there was not enough time to reheat.

^d^Missing time-series mass flow rate data.

Chilldown time was determined as follows: In practice, the most stringent chilldown criteria would be determined from a measured stream temperature downstream of the test section reading lower than the saturation temperature based on the downstream pressure; however, this measurement was not available for the current tests. Based on boiling heat transfer theory, nucleate boiling would end when the inner surface temperature drops below that of the onset-of-nucleate boiling (ONB). As a result, the wall heat flux would switch from higher boiling heat flux to much lower single-phase convective heat flux that would reflect a change on the outer wall surface temperature gradient with time. A computed inner wall temperature could also not be used to determine end of chilldown; while the inverse conduction method of Burggraf^39^ can be used to determine inner wall temperature for bare tubes, due to the unknown thermal contact resistances between the coated layer and tube inner wall as well as among adjacent coated layers, inner wall temperature could not be determined for coated tubes. Therefore, outer wall temperature data had to be used to determine end of chilldown. Three chilldown criteria were explored: (1) the averaged exit outer wall temperature was compared to the liquid saturation temperature (based on local downstream pressure), (2) the first derivative of outer wall temperature (with respect to time) reaching and remaining near 0 K/s (due to minimal convective heat transfer between single-phase liquid and tube), and (3) a peak value in the second derivative of outer wall temperature (with respect to time) which would indicate the slope change in the chilldown curve occurring at onset of nucleate boiling (ONB). The first method was found to be unreliable due to inaccurate chilldown time estimations attributed to the significant difference between inner and outer wall temperature at higher layers of coating. The third method also yielded inaccurate chilldown time estimations attributed to the absence of a true global maxima in the second derivative at higher layers of coating. Therefore, the second method using the first derivative (typically using test section averaged temperature), slightly conservative but consistent across all scenarios, was used to determine the end of chilldown in all test cases.

Chilldown mass was the total consumed LN_2_ mass at the end of chilldown as read by the flow meter downstream of the test section:

1 $$m_{LN2} = {\int}_0^{t_{end}} {\dot mdt}$$

where *t*_end_ is the end of chilldown time and $\dot m(t)$ is the time-dependent LN_2_ mass flow rate measured by the gas flow meter. Steady state Reynolds (Re) number (defined at the end of chilldown when single phase liquid flow is established) and mass flux were evaluated using inner diameter and saturation conditions based on the measured test section pressure:

2 $${\rm{Re}} = \frac{{4\dot m}}{{\pi D\mu }}$$

For uncoated tubes, the method of Burgraff^39^ was used to determine inner wall temperature and transient radial heat conduction through the tube as follows:

3 $$\begin{array}{l}q^{{\prime}{\prime}}_w = \rho c_P\left( {\frac{{r_i^2 - r_o^2}}{{2r_i}}} \right)\frac{{dT_o}}{{dt}} + \frac{{\left( {\rho c_P} \right)^2}}{k}\left( {\frac{{r_i^3}}{{16}} - \frac{{r_o^4}}{{16r_i}} - \frac{{r_o^2r_i}}{4}\ln \left( {\frac{{r_i}}{{r_o}}} \right)} \right)\frac{{d^2T_o}}{{dt^2}}\\ \qquad+ \frac{{\left( {\rho c_P} \right)^3}}{{k^2}}\left( {\frac{{r_i^5}}{{384}} - \frac{{3r_o^4r_i}}{{128}} + \frac{{3r_o^2r_i^3}}{{128}} - \frac{{r_o^6}}{{384r_i}} - \frac{{r_o^2r_i^3}}{{128}}\ln \left( {\frac{{r_i}}{{r_o}}} \right) - \frac{{r_o^4r_i}}{{32}}\ln \left( {\frac{{r_i}}{{r_o}}} \right)} \right)\frac{{d^3T_o}}{{dt^3}}\end{array}$$

where $q^{\prime\prime}_w$ is the radial heat flux through the tube, *ρ*, *C*_*P*_, and *k* are the tube density, specific heat, and thermal conductivity, respectively, *r*_*i*_ and *r*_*o*_ are the inner and outer radii, and *T*_*o*_ is the outer wall temperature. Heat transfer coefficient was then computed as follows:

4 $$h_{\rm{quench}} = \frac{{q_w^{\prime\prime} + q_{\rm{axial}}^{\prime\prime} + \frac{{r_o}}{{r_i}}\left( {q_{\rm{rad}}^{\prime\prime} + q_{\rm{solidcond}}^{\prime\prime} + q_{\rm{gascond}}^{\prime\prime} } \right)}}{{T_i - T_{\rm{sat}}}}$$

where $q_{axial}^{\prime\prime}$ is the axial conduction along the tube; the terms in parenthesis are the radiation, solid conduction, and gaseous conduction parasitic heat leak terms, respectively, *T*_*i*_ is the inner wall temperature which comes from Burgraff’s method, and *T*_sat_ is the saturation temperature based on the measured pressure. The method to calculate the different heat fluxes in Eq. 4 has been shown in many other papers, see for example ref. ^22^ and ref. ^27^.

### Governing physics of chilldown

**Fig. 1:**
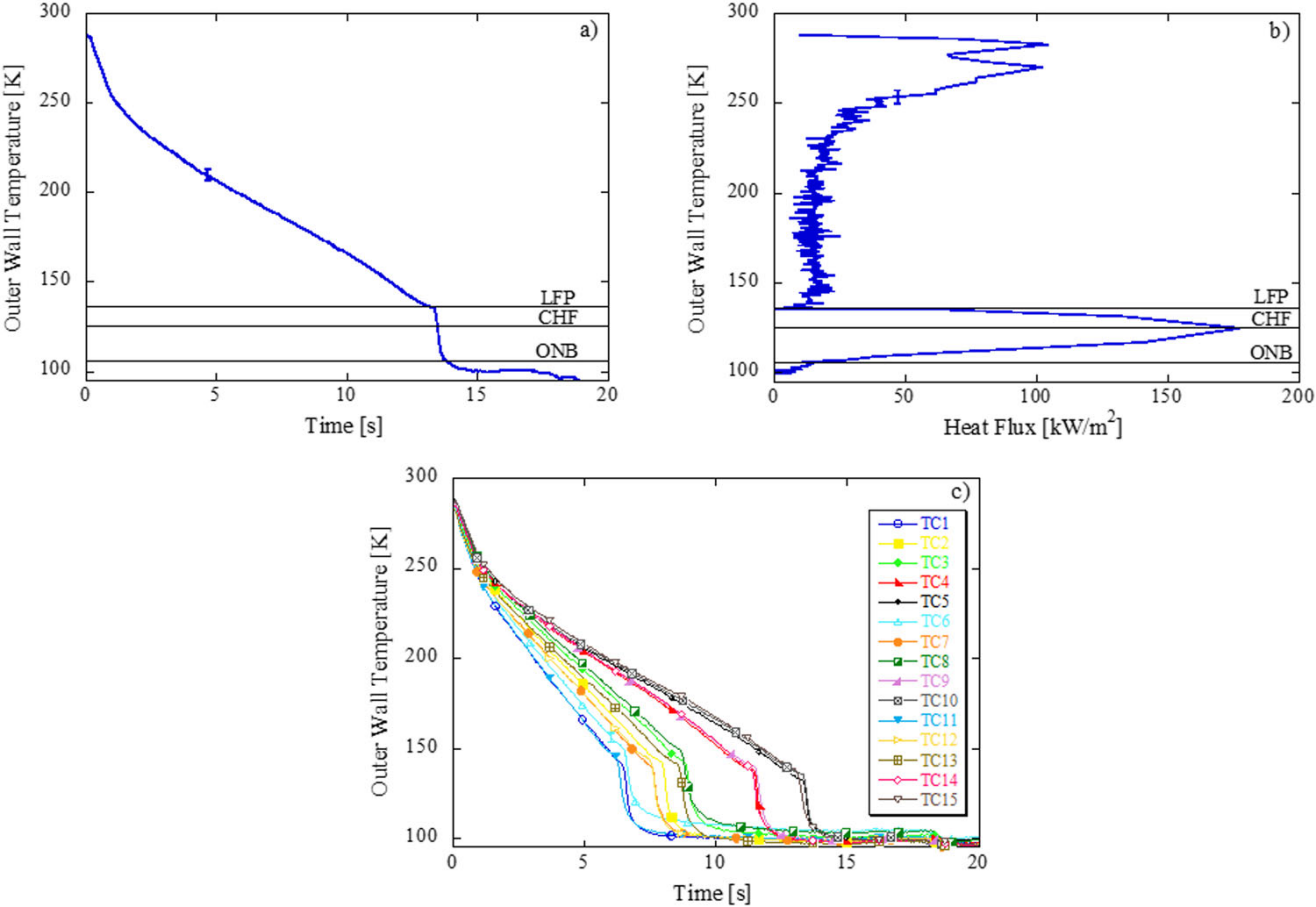
Test F2 (Flight_Day1_2, continuous flow, 550 kPa source pressure, 67324 Re, bare tube surface, 0.05 G level (g/g0)). **a** Chilldown curve based on average exit wall temperature, **b** Boiling curve based on average exit wall temperature, **c** Chilldown curve of all TCs.

Figure 1a shows the chilldown curve of averaged exit wall temperature (TC5, TC10, TC15) and Fig. 1b shows the boiling curve based on the averaged exit wall temperature in microgravity. Averaging was done by adding the temperatures and dividing by the number of sensors. Figure 1c illustrates the chilldown curve of all thermocouples (TCs) placed on the tube outer wall according to Fig. 5d, e in the “Methods” section. Errors bars are plotted but barely discernable. Three boiling regimes, film boiling (FB), transition boiling (TB), and nucleate boiling (NB), and single-phase convection are separated by three critical points, the Leidenfrost Point (LFP), Critical Heat Flux (CHF), and the onset of nucleate boiling (ONB). The chilldown curve begins in the film boiling regime where the cold liquid entering the warm tube experiences violent boiling. Depending on the local conditions, the flow will proceed into dispersed flow FB (high quality, low subcooling, low mass flux) or inverted annular FB (low quality, high subcooling, high mass flux)^40,41^. The high wall surface temperature causes the liquid to completely vaporize before reaching the surface resulting in an inner liquid core and outer annular vapor core. This vapor blanket along the wall insulates the warm pipe from the cold liquid, causing the temperature of the pipe to decrease, albeit slowly. FB is the least efficient quenching mechanism. As the transfer line chills down, the system approaches the LFP, or rewet temperature, where heat flux is at a minimum (during boiling). Heat transfer here is a minimum due to the inefficiency of heat transfer between cold vapor and wall. LFP is also characterized by the onset of a rapid drop in wall temperature. As shown in Fig. 1c, the LFP occurs at later times for TCs located farther downstream. This trend demonstrates the location of the quenching front as it propagates downstream as chilldown evolves. The flow then proceeds and passes quickly through TB, characterized by intermittent liquid contact along the walls. TB ends when liquid is in full contact with the walls at the point of CHF. Heat transfer is a maximum at CHF due to the highly efficient cooling process of boiling. Nucleate boiling follows, where heat is transferred by vapor bubbles formed in surface cavities that are swept away from the tube surface. Depending on the inlet conditions, NB can be liquid-convection dominate or nucleation-dominate^31^. As the wall cools further, the tube inner surface approaches the ONB, characterized as the point at which the system evolves from nucleate two-phase cooling to single-phase liquid convection and an obvious slope change in the chilldown curve. Vapor-free liquid marks the end of the chilldown test. The single-phase cooling causes the wall temperature to drop slowly to the liquid saturation temperature and then remain steady as heat transfer reduces to near zero. In microgravity, circumferential TCs at each station have almost identical chilldown behavior at any axial distance from the inlet; stratification effects normally seen for horizontal tubes in 1-g disappear, leading to axisymmetric flow patterns through the tube, and thus uniform chilldown circumferentially.

### Bare vs. coated tube in microgravity, continuous flow

**Fig. 2:**
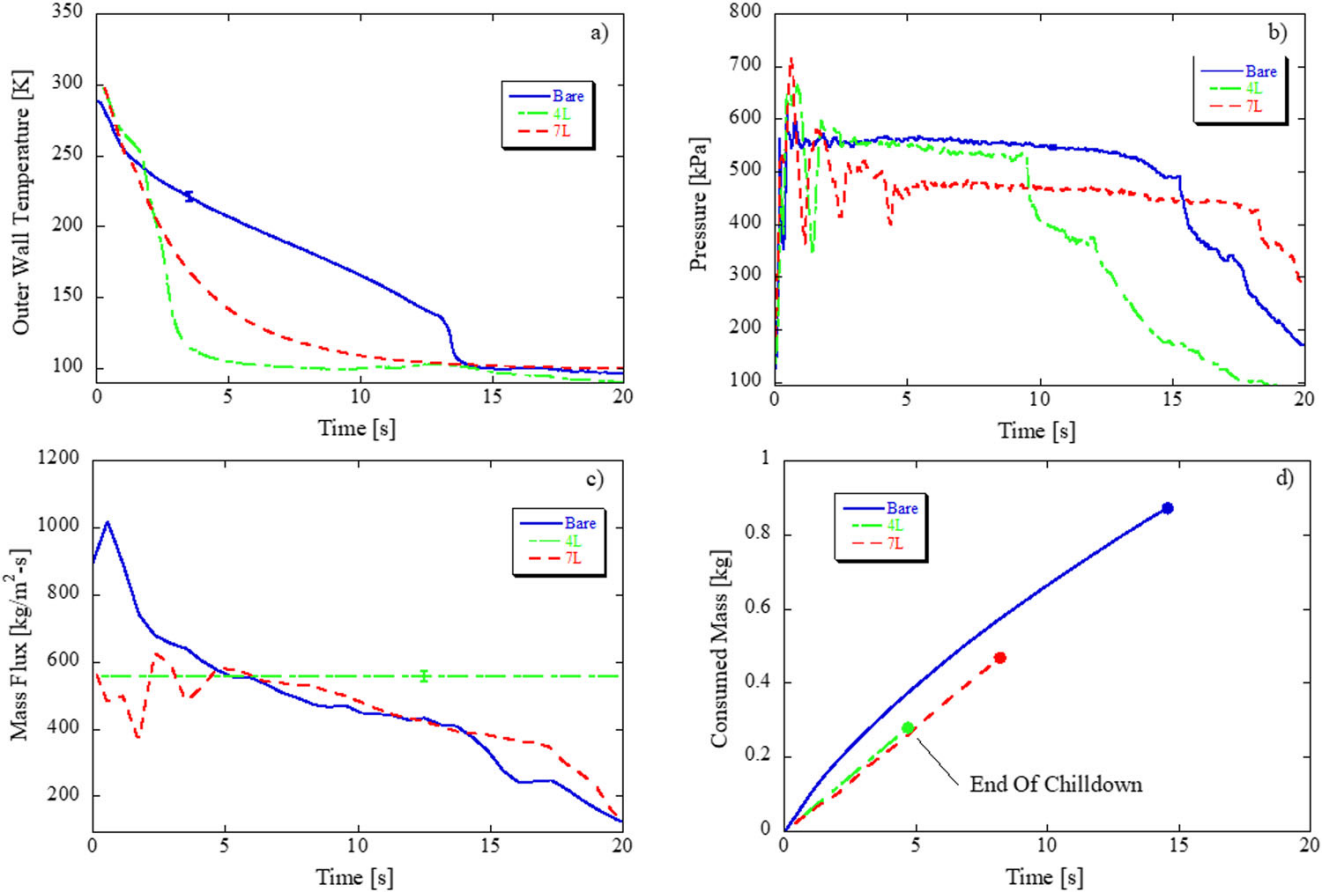
Effect of teflon coating thickness in microgravity: 550 kPa source pressure, continuous flow, 0.05 G level (g/g0). F2 (Flight_Day1_2, 67324 Re, bare surface) versus F22 (Flight_Day4_1, 74196 Re, 4 L coating) versus F13 (Flight_Day3_1, 63912 Re, 7 L coating) **a** Chilldown curve, **b** exit pressure, **c** mass flux, and **d** total propellant mass consumed based on averaged exit wall temperature.

Figure 2a–d plot chilldown curves, exit pressure, mass flux, and total consumed liquid mass for bare, 4 L, and 7 L coated tubes for higher steady state Re (63,912–74,196). The initial fluctuations in pressure measurements in Fig. 2b are due to the transient nature of the flow at start of the test. Shortly after the transient start, downstream pressure measurements reach their steady-state value and remain there until at least the end of chilldown in all three cases. The mass flux of the 4 L coating case in Fig. 2c is a straight horizontal line because of missing timed mass flow rate data for that run; a linear correlation was developed between averaged inlet pressure and averaged mass flow rate for cases with available mass flow rate data that were run at 0.05 g level and were completed under one parabola. This linear correlation was then used to calculate an average mass flow rate for cases with missing mass flow rate data (but available inlet pressure data).

Trends are as follows: First, coating the inner wall of the tube drastically affects the chilldown behavior and leads to faster chilldown times. The low thermally conductive Teflon layer acts as an insulator between cold fluid and warm wall; the inner surface temperature chills down quickly without cooling the entire tube mass. The lower inner wall surface temperature earlier on means that the Leidenfrost point is reached faster such that the liquid can stay in contact with the tube for the heat transfer to be in TB and NB that reduces the poor heat transfer film boiling time; this is substantiated by the drastic slope change for 4 L and 7 L tube indicating the LFP is reached earlier on relative to the bare tube. Note that Fig. 2a plots outer wall temperature; the actual inner wall temperature for the coated tubes will be significantly lower since the coating restricts the heat transfer between inner and outer walls. Second, less mass is consumed for coated over bare tubes as substantiated in Fig. 2d; the 4 L and 7 L coated cases have 68 and 46% propellant mass savings over the bare tube, respectively.

Third, however, there is an apparent point of diminishing returns; this trend of improved chilldown performance upon addition of coating is reversed when the number of coating layers is increased from 4 to 7 because the chilldown time is faster for 4 L (4.7 s) compared to 7 L (8.2 s) case. Similarly, from 4 L to 7 L, the propellant mass savings and chilldown efficiency are reduced. This crossover in performance and possible existence of an optimal coating layer is explained by counteracting heat transfer mechanisms: (1) the low thermal conductivity of the coating layer facilitates the faster temperature drop of tube inner surface by restricting heat transfer between inner surface and bulk of the metal tube and (2) the low thermal conductivity coating also creates a thermal resistance that restricts the heat conduction between bulk of the tube and cooling fluid. With these contrasting mechanisms at play, the thickness of the coating must be such that it is thick enough to quickly lower the tube inner surface temperature while being thin enough to facilitate fast wall chilldown. However, the presence of the coating accelerates chilldown as evident in any comparison between bare and coated tube at similar thermodynamic conditions.

### Continuous versus pulse flow in microgravity, bare tube

**Fig. 3:**
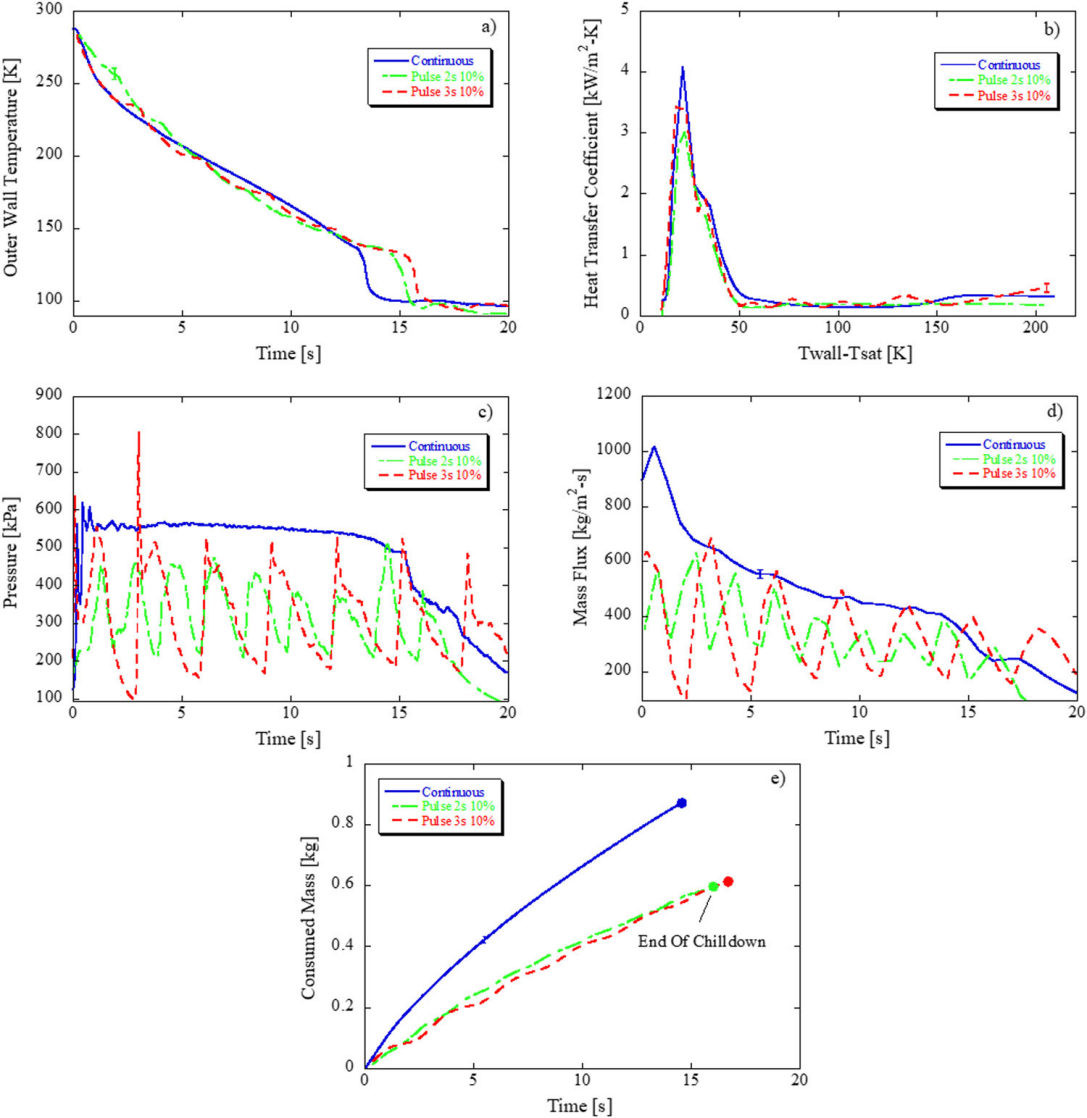
Effect of pulse flow on a bare tube in microgravity: 550 kPa source pressure, bare tube surface, 0.05 G level (g/g0)—F2 (Flight_Day1_2, 67324 Re, continuous) versus F3 (Flight_Day1_3, 53859 Re, pulse 2 s 10%) versus F4 (Flight_Day1_4, 64092 Re, pulse 3 s 10%). **a** Chilldown curve, **b** Heat transfer coefficient versus wall superheat, **c** Exit pressure, **d** mass flux, and **e** total propellant mass consumed based on averaged exit wall temperature.

Figure 3a–e plot chilldown curves, heat transfer coefficient, pressure, mass flux, and total consumed liquid mass for continuous flow and pulsed flow at a period of 2 s and duty cycle 10% (valve on 0.2 s, valve off 1.8 s) and for period 3 s and duty cycle 10% (valve on 0.3 s, valve off 2.7 s) at higher Re (53859–64092). Trends are as follows: First, both continuous and pulse flow exhibit the same chilldown curve and proceed through the same transition points. For pulse flow, the longer the valve-off time, the more the tube temperature stabilizes as residual cooling due to blowdown diminishes. Second, from Fig. 3a, e, it is clear that pulse flow achieves chilldown using less propellant but at the cost of longer chilldown time due to better use of sensible and latent energy of the fluid. Figure 3c, d shows fluctuations in pressure and mass flux that are due to valve cycling, that these fluctuations continue until end of chilldown, and that the fluctuation amplitudes are higher for longer periods. From Table 1, there is 29–32% mass savings with pulse flow in comparison to continuous flow at these flight conditions. Third, for a fixed duty cycle, reducing the valve-open time leads to slightly shorter chilldown times (although not shown directly in Fig. 3) and, slightly less propellant consumption as shown in Fig. 3a, e; this trend compares well with previous pulse flow tests for both LN_2_^33^ and LH_2_^37^. Fourth, for this particular comparison, Fig. 3b shows that continuous flow exhibited a higher CHF over pulse flow, and that reducing the valve open time reduced the CHF. Because of the temperature stabilization when the valve was cycled off, the temperature does not drop as rapidly in pulse compared to continuous flow which caused the wall temperature first derivative term to be lower at CHF in pulse flow. However, if the CHF was traversed when the valve was on, it is expected that the pulse flow heat transfer coefficient (HTC) would be nearly equivalent to that of continuous flow. For bare tubes in microgravity, while higher frequency, shorter pulse widths are favorable from a chilldown efficiency standpoint, more valve cycles implies higher risk of valve degradation and potential failure. Therefore, there is an inherent trade-off in which the optimal valve duty cycle could be determined.

### Performance gain of combined pulsed flow and coated tubes in microgravity

**Fig. 4:**
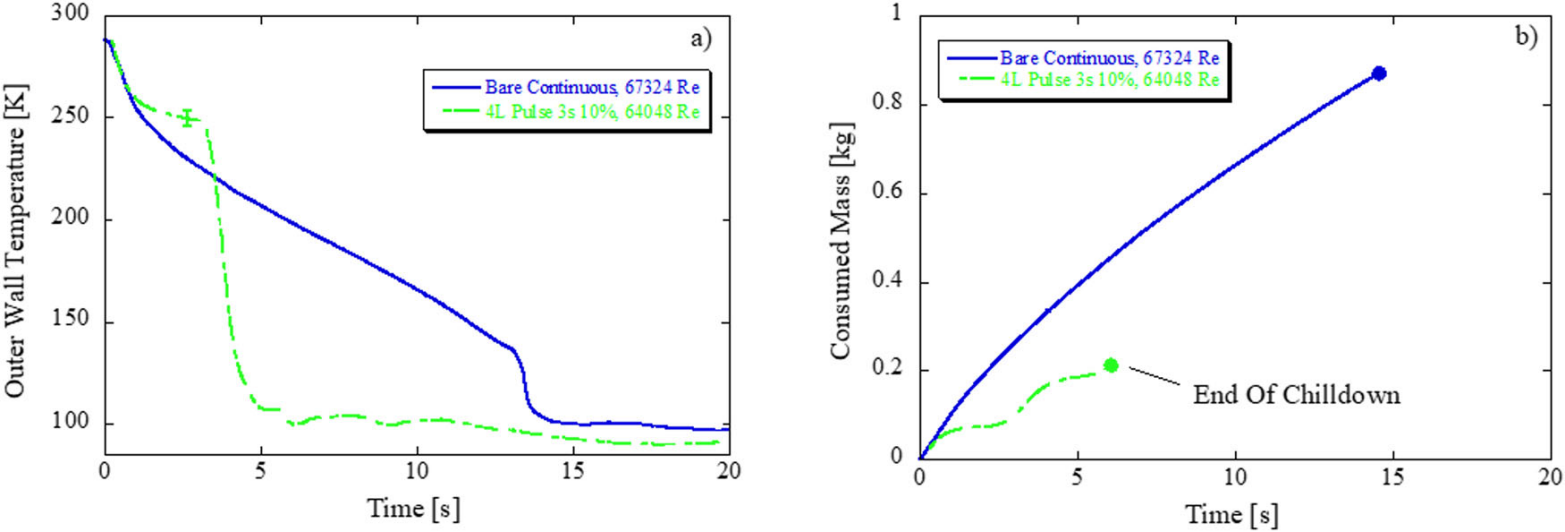
Combined effect of coatings and pulse flow in microgravity: 0.05 G level (g/g0)—F2 (Flight_Day1_2, 550 kPa source pressure, 67,324 Re, bare surface, continuous) versus F28 (Flight_Day4_7, 550 kPa source pressure, 64,048 Re, 4 L Coating, 3 s 10%). **a** Chilldown curve and **b** total propellant mass consumed based on averaged exit wall temperature.

Figure 4a, b plot chilldown curves and total consumed liquid mass for bare tube with continuous flow and 4 L coated tube with pulsed flow characterized by a period of 3 sec and duty cycle of 10% (valve on 0.3 s, valve off 2.7 s) at higher Re (64,048–67,324). Trends are as follows: First, the effect of coating on reducing chilldown time seems to outweigh the effect of pulse flow on increasing chilldown time as evidenced by the sharp drop in temperature at ~4 s in Fig. 4a for the coated tube. Second, the individual benefits of propellant mass savings with coating and pulse flow are nearly perfectly superimposed, leading to a 76% reduction in propellant consumption. Results thus show that high performance is still achieved in microgravity for pulse flow with a low thermal conductivity coating which leads to a reduction in chilldown time and mass and increase in chilldown efficiency over continuous flow with a bare tube.

## Methods

### Experimental description

**Fig. 5:**
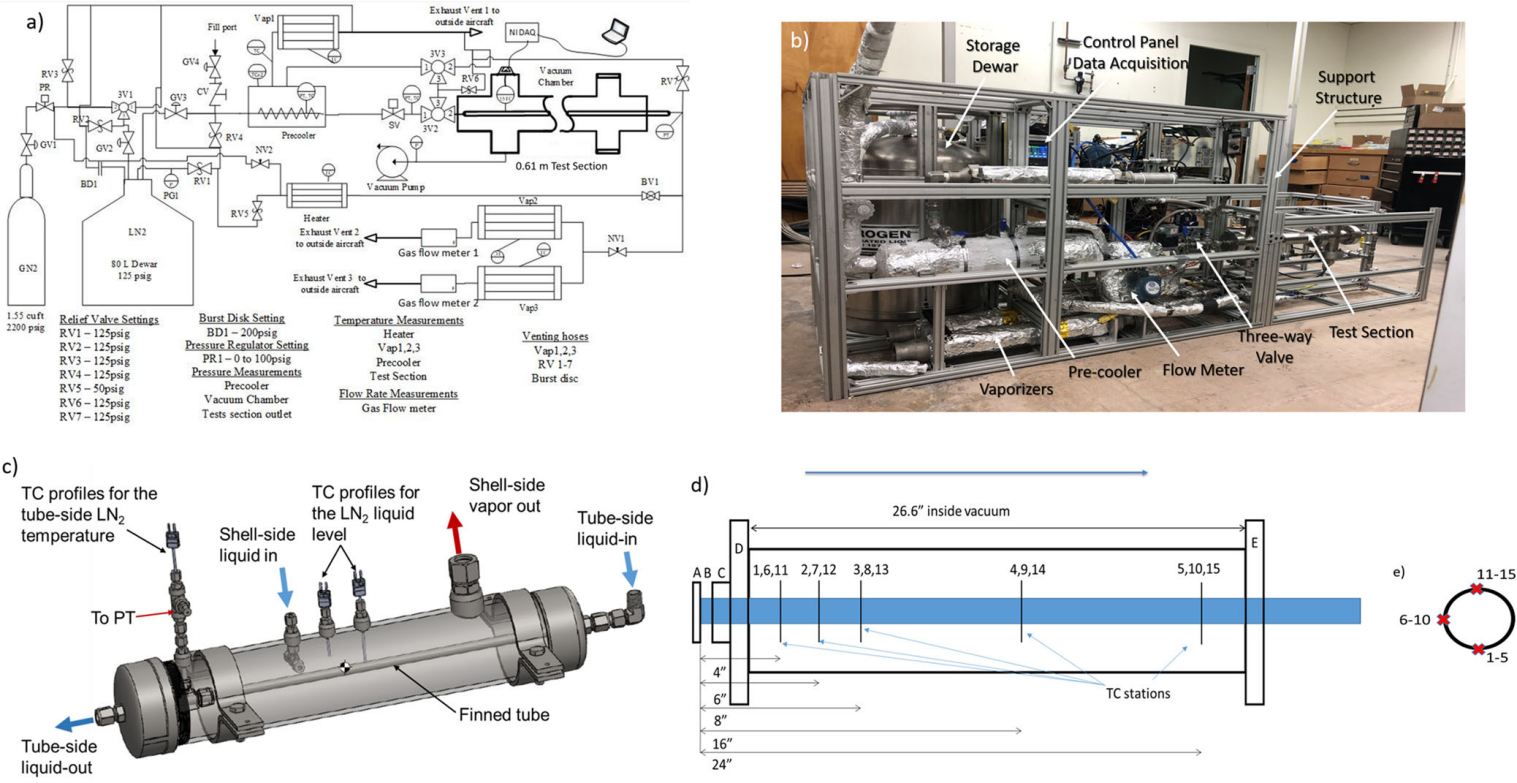
Experimental Design. **a** Flight system piping and instrumentation diagram, **b** actual flight line chilldown rig, **c** pre-cooler, **d** test section, vacuum chamber, and thermocouple locations, A: Inlet, B: 3.81 cm (1.5 in) long tube section, C: ultra-torr fitting, D: left flange, E: right flange and **e** thermocouple locations at each station.

The authors have completed four successful cryogenic line chilldown parabolic flight campaigns including the current campaign between 2015 and 2020 and are familiar with system designs, troubleshooting, issues, and failures that arise with microgravity flight testing. The fourth-generation system was modified based on flights from the first-^8^ and second-generation^36^ systems. As before, the system is intended for both ground and flight experiments. Figure 5a shows a system flow network and piping and instrumentation diagram while Fig. 5b shows a picture of the actual flight rig.

LN_2_ was supplied to the system from an 80-liter vacuum-jacketed dewar, with a relief valve set at 861 kPa. A gaseous nitrogen (GN_2_) cylinder initially pressurized at 15 MPa was used to pressurize the dewar to a set value for each test, which ranged between 90 and 830 kPa absolute pressure. Dewar pressure was managed by a pressure regulator that controlled the dewar pressure to within 35 kPa of the set value during each test. Depressurization was carried out by opening the globe valve 2 (GV2) and the three-way ball valve 1 (3V1) to allow ullage gas to vent to the atmosphere.

The dewar was used to supply the LN_2_ both for prechilling the plumbing upstream of the test section and for conducting the actual chilldown experiment. LN_2_ was delivered through valve GV3 that was connected through a 1.2 m long, 1.27 cm outer diameter (OD), 1.18 cm inner diameter (ID) 304 (stainless steel) SS braided hose to the inner tube of the precooler (or subcooler) shell-tube heat exchanger shown in Fig. 5c. The subcooler served three purposes: (1) to preserve subcooling of the liquid from the storage tank flowing through the transfer line by eliminating parasitic heat leak, (2) to slightly subcool the LN_2_ in the transfer line, since the saturation temperature of the shell side was always lower than the tube side, and most importantly (3) to ensure single-phase liquid at the inlet of the test section. The liquid level of the nitrogen pool was monitored by three thermocouples (TC) inside the subcooler, two at the shell-side and one at the outlet to Vap1. The temperature readings of these TCs were displayed on a laptop in real-time. The level of the LN_2_ pool was inferred from the TC insertion depth. A 2.5 cm ID port allowed evaporating liquid to escape the subcooler. The fluid was directed to an electrically heated “vaporizer” Vap1 which vaporized any entrained liquid and warmed the vapor to above 273 K before entering the atmosphere. Two layers of 6.35 mm thick aerogel insulation were wrapped around GV2, the hose upstream of the subcooler, the subcooler itself, 3V2, 3V3, and the 3 cm length of tube between 3V3 and the subcooler to minimize heat leak into the system upstream of the test section.

During the prechilling process, the liquid exiting the inner tube of the subcooler was directed by two “T-type” 316SS 1.27 cm ID three-way ball valves (3V3 and 3V2) to a fill-port on top of the outer vessel of the subcooler. A 3 cm long, 1.270 cm OD, and 1.168 cm ID 304SS tube connected 3V3 to the subcooler. A pressure transducer and TC labeled “PT”, “TC” in Fig. 5a were placed between a solenoid valve (SV) and a three-way vale (3V2) at a distance of 7 cm from the downstream side of the inner tube of the subcooler to measure the fluid pressure and fluid temperature. This station was also used to determine the thermodynamic state of the fluid at the inlet of the test section. Once the flow inlet temperature reached a steady value, and that steady temperature was below the saturation temperature based on the measured pressure, a chilldown test was ready to commence. As shown in Fig. 5a, the test section was enclosed in the vacuum chamber and sealed by two flanges (D and E). A 316SS vacuum chamber was used to reduce radiation and gas conduction parasitic heat leak to the test section from the surroundings, which reduces the uncertainty in the calculation of wall-to-fluid heat flux. A mechanical pump reduced background pressure to ~1 Pa.

The needle valve downstream of the test section (NV1) was used to provide fine-tuning of the mass flow rate so that tests could be run at different flow rates for the same dewar pressure setting. The flow was routed from the needle valve by a SS tube to two separate vaporizers (labeled Vap2 and Vap3) that were electrically heated to vaporize the liquid-vapor two-phase flow. To enhance the heat transfer in the vaporizer, eight 1.27 cm OD copper tubes were packed inside the vaporizer in an octaweb configuration. One electrical heating tape was wrapped around the vaporizer to heat it to 550 K before each test. A TC was placed on the outer surface of each heating tape to monitor the temperature in real time. The flow out of Vap2 and Vap3 entered two separate, identical gas flow meters (Gas Flow Meters 1 and 2) that each had a capacity of 3000 standard liters per minute. The flow was then directed to the airplane vent ports downstream the flow meters.

### Test sections

Three, 0.914 m (36 in) long, 0.051 cm wall thickness, 1.27 cm outer diameter SS304 (properties taken from^42^) test sections were individually flight tested: a bare tube with no coating, and a tube with a 4 layer and 7 layer coating. For the coated tubes, the SS tube was coated with low-thermal conductivity thin Teflon layers on the inner surface. Specifically, the coating material was made of Fluorinated Ethylene Propylene (FEP) produced by DuPont and classified by DuPont as Teflon 959G-203 that is a black color paint. The coating was applied by using a pour and drain process. After each pour and drain, the fresh film layer was cured in a furnace through a standard sintering procedure before adding another layer by the same pour and drain procedure. As a result, the final thickness of the coated layer depends on the total number of layers processed; for example, the 4 L coating went through the pour and drain process four separate times. To measure the coating layer thickness, high resolution computer tomography x-ray scans of the tube cross sections were obtained using a Phoenix v|tome|x M system in the Nano Research Facility at the University of Florida. Scanning was carried out using a 240 kV X-ray tube and a tungsten-on-beryllium target, with the following settings: 200 kV, 50 milliamps, and 0.5 mm Tin filter. Images were collected from 1600 pixels horizontal, 2024 pixels vertical, 0.5 s detector exposure, averaging of 4 images per rotation position with a one-exposure skip and a total of 2200 rotational positions. The average thickness per layer was ~15.12 µm and the uncertainty for each layer was $\pm$0.7 µm.

### Instrumentation and data acquisition

Next, for data acquisition (DAQ) and instrumentation, a Labview Virtual Instrument software and National Instrument (NI) CompactDAQ hardware was used to collect all sensor data to be displayed in real-time on a laptop. The sampling rate of all the sensor measurements was set to 16 Hz. Two NI-9214 TC modules read the signals from all the T-type TCs. NI 9205, an analog input module, read all the voltage signals from pressure transducers. The Labview VI controlled the opening and closing of the solenoidal valves (SVs), through a combination of NI-USB 6009 and Solid-State relay. In the case of continuous flow, the relay energized the solenoid valve after receiving a constant voltage signal. For pulse flow, the relay energized and de-energized SV according to a rectangular waveform voltage signal generated by the Labview VI. Signals of the two mass flow meters (Alicat M3000 - SLPM) downstream of the vaporizers were read by the program directly without the NI DAQ system.

Fifteen TCs were soldered to the outside of each tested tube. Five stations were spaced out axially in Fig. 5d and three TCs were spaced out radially 90^o^ (top, bottom, side) at each station as shown in Fig. 5e. Two cryogenic rated PTs were placed near the inlet and after the outlet of the test section by yor-lok fittings, respectively to provide the transient pressure histories at the two locations. The rest of the instrumentation is shown in Fig. 5a.

### Uncertainty analysis

Root-sum-square uncertainty analysis was conducted in a similar fashion as in refs. ^27,29^; uncertainties for test section dimensions, vacuum chamber dimensions, and thermal properties were similar as in^29^. Standard error propagation rules were applied to compute uncertainties in chilldown time (2.1%), propellant mass consumed at steady state (2.5%), mass flux (2.8%), and Re number (3.3%). The median relative uncertainties were 8–10% in Burggraf heat flux, total heat flux, and HTC, and 25% in parasitics across all the bare tube cases. The number of outliers in relative uncertainties were on the order of 10^1^ or fewer in each case and occurred post-chilldown. Therefore, the 95% quantile accurately represents the maximum relative uncertainties in Burgraff heat flux, parasitics, total heat flux, and HTC which are reported in Table 1 and depicted as error bars in plots.

### Experimental methodology

The experimental methodology to conduct a test was as follows: At the start, the needle valve was set to the target position to set test section pressure, Vap 1, Vap2, and Vap3 were heated up to 550 K, and the vacuum pump system was turned on. The total time from engaging the pump until reaching 1 Pa inside the vacuum chamber was ~15 min. Concurrently, the inner tube inside the subcooler was chilled by pressurizing the dewar, opening GV2, and directing the flow through 3V3 and 3V2 to the fill port of the subcooler. The subcooler took ~10 min to completely chill and fill. Then, 3V2 was shut off to stop the flow from 3V3, and the supply dewar was pressurized by opening the pressure regulator to the desired gauge pressure for the dewar. Pressurization was done as quickly as possible before the liquid inside the dewar could re-saturate at the new dewar pressure, and also before the liquid inside the plumbing upstream of the test section could gain enough heat to start boiling. Shortly after, 3V3 was turned to start the flow into the test section to begin a chilldown test. Once the TC readings dropped below the saturation temperature and maintained a steady temperature, GV2 and SV was closed. This marked the end of the test. In preparation for the next test, Heater 1 was turned on, and both 3V2 and 3V3 were rearranged so that warm gas could enter the test section to carry out the reheat process. After reheating was finished, NV1 was set at the new position. Vap2 and Vap3 were allowed to heat up to above 550 K, and the subcooling process was repeated to account for the lost LN2. At this time, the system is ready for another run.

### Reporting summary

Further information on research design is available in the Nature Research Reporting Summary linked to this article.

## Discussion

**Fig. 6:**
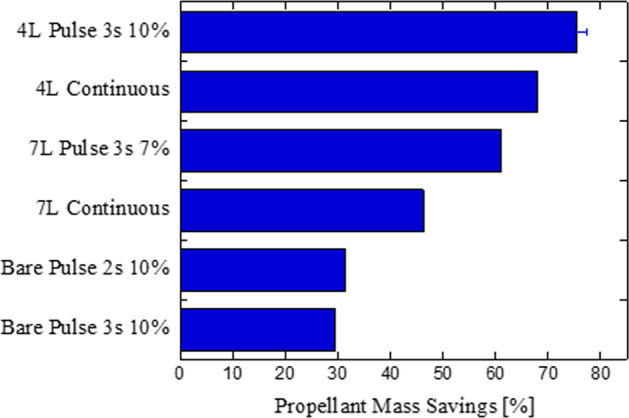
Performance gains of pulse flow and coated tube in microgravity: 550 kPa. Source pressure, 0.05 G level (g/g_**0**_)—F2 (bare continuous) vs F3 (bare pulse 3 s 10%) vs F4 (bare pulse 2 s 10%) vs F13 (7 L continuous) vs F14 (7 L pulse 3 s 7%) vs F22 (4 L continuous) vs F28 (4 L pulse 3 s 10%)—propellant mass savings as compared to base case of Flight_Day1_2 (continuous flow and bare tube).

Figure 6 summarizes results in terms of mass savings for the cases discussed previously. Overall, pulse flow through a coated tube significantly outperforms continuous flow through a bare tube at any flow rate under microgravity. The combined case of pulse flow and coated tube also outweighs the performance gains of just coated tube or pulse flow. Across a wide range of Reynolds numbers, results show that the combination significantly enhance performance, with a reduction in consumed mass up to 75% relative to continuous flow for a bare transfer line. Surprisingly, when compared to 1-g coated tube pulse flow tests from ref. ^33^, at somewhat similar inlet pressure, period, and duty cycle, the mass savings in going from continuous flow with a bare tube to pulse flow with a coated tube is slightly higher in microgravity (~75%) versus in 1-g (67%) at similar high Re. The lower mass savings in 1-g can easily be attributed to the fact that the duty cycle of the 1-g coated tube pulse flow test is 20% compared to 10% of 0-g coated tube pulse flow test in the current work. Lower duty cycle is predicted to increase mass savings^33^ which means that a 1-g coated tube pulse flow test performed at 10% duty cycle would have >67% propellant mass savings. At high Re, the mass savings would be roughly equal in microgravity and 1-g because of forced convection dominating over buoyancy effects. However, at low Re, 1-g results would be expected to yield higher mass savings due to the aforementioned lack of buoyancy-assisted cooling in microgravity at low Re, whether comparing 1-g pulse flow to 0-g pulse flow or 1-g coated tube flow to 1-g coated tube flow. Therefore, with optimization of coating thickness and pulse characteristics performed a priori, coated tube and pulse flow can be used for transfer line chilldown to significantly save chilldown time and mass for all future in-space cryogenic transfers.

## Supplementary information

Reporting Summary

## Data Availability

Supplementary information accompanies the paper on the *npj Microgravity* https://www.nature.com/npjmgrav/.
